# Dietary Prebiotics Modulate Omeprazole‐Induced Alterations in the Gut Microbial Signature

**DOI:** 10.1002/mnfr.70307

**Published:** 2025-10-30

**Authors:** Molly Jenee Buccola, Adhini Kuppuswamy Satheesh Babu, Henry A. Paz, Nizhoni Dawn Porter, Harini Srinivasan, Rachel Lyn Ricks, Keaton Rosquist, Jose Luis Torres, Ying Zhong, Thunder Jalili, Umesh D. Wankhade, Pon Velayutham Anandh Babu

**Affiliations:** ^1^ Department of Nutrition and Integrative Physiology, College of Health University of Utah Salt Lake City Utah USA; ^2^ Arkansas Children's Nutrition Center University of Arkansas for Medical Sciences Little Rock Arkansas USA; ^3^ Department of Pediatrics University of Arkansas for Medical Sciences Little Rock Arkansas USA

**Keywords:** blueberries, gut microbiome, omeprazole, prebiotics, proton pump inhibitors, strawberries

## Abstract

Proton pump inhibitors (PPIs) are commonly used to treat heartburn and acid‐related disorders. However, their misuse and prolonged use contribute to gut dysbiosis. This study investigated whether well‐known prebiotic dietary sources, blueberries or strawberries, can reverse PPI (omeprazole) induced dysbiosis and gut inflammation by modulating gut microbes. Male C57BL/6J mice (7 weeks old) were fed a diet with or without omeprazole (40 mg/kg diet), blueberry (3.7% in the diet; ∼1.5 human servings) or strawberry (2.35% in the diet; ∼2 human servings) for 12 weeks. Metabolic parameters, gut microbes (in the cecum and colon), and inflammatory markers were assessed. In this study, no changes were observed in metabolic parameters in mice fed a diet supplemented with omeprazole or berries. Second, blueberry or strawberry supplementation at nutritional dosages improved alterations in gut microbial ecology induced by omeprazole, with effects varying between the cecum and colon. Third, strawberry supplementation reduced omeprazole‐induced gut inflammation. Fourth, selected genera were either positively or negatively associated with markers of gut inflammation, suggesting that dietary berries can ameliorate inflammatory signaling through modifications in the gut microbiome. Dietary berries represent a potential nutritional strategy for improving PPI‐induced gut dysbiosis and inflammation.

## Graphical abstract

Dietary prebiotic sources such as blueberries and strawberries may help improve proton pump inhibitor (PPI)‐induced gut dysbiosis. These berries represent a promising nutritional strategy for mitigating PPI‐associated gut dysbiosis and inflammation.

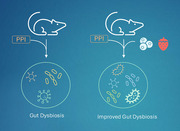

**Abbreviations**
CDI: *Clostridioides difficile* infections
PPI: proton pump inhibitors

## 1 Introduction

Proton pump inhibitors (PPIs) are the most prescribed medications for treating heartburn and acid‐related disorders. Evidence shows that 25% of the adult population globally is prescribed PPI, underscoring the extensive use of these medications [1]. PPIs reduce acid production in gastric parietal cells by forming disulfide bonds with H^+^/K^+^‐ATPase pumps to combat reflux disorders. The most common PPIs include omeprazole, esomeprazole, pantoprazole, lansoprazole, and rabeprazole. Omeprazole ranks as the most frequently prescribed medication in England and the eighth in the United States [1]. However, PPIs are sometimes overprescribed by doctors and misused by patients, as the prescriptions are not always required [2, 3].

PPI use is considered safe in the short‐term, but long‐term usage is linked to several complications [1]. PPI can alter gut microbial ecology and contribute to dysbiosis in the gastrointestinal tract [4]. Prolonged PPI usage can predispose individuals to small intestinal bacteria overgrowth [5]. By raising gastric pH, PPI can increase the gastric bacteria count to 1000‐fold [6]. Through various mechanisms, including but not limited to dysbiosis, PPIs have been associated with adverse cardiac events, renal impairment, and worsened mortality in cirrhosis patients, neurodegenerative diseases, and pancreatic dysfunction [7, 8, 9, 10, 11]. Additionally, PPIs are linked to *Clostridium difficile* overgrowth, which can lead to potentially life‐threatening diarrhea [12]. They can also exacerbate intestinal permeability in colitis and irritable bowel disease [13]. Intestinal microbes, particularly *Bacteroides*, *Bifidobacterium*, and *Enterococcus*, are essential for synthesizing several B vitamins and vitamin K2 [14]. PPI users often experience calcium, magnesium, iron, Vitamin D and B12 deficiencies, especially those who overuse them [14]. This may result from small intestinal bacterial overgrowth, dysbiosis‐related poor nutrient absorption, or reduced vitamin‐synthesizing commensal bacteria [14]. Given the widespread use of PPI globally, the complications associated with PPI usage must be addressed. PPI‐induced gut dysbiosis could be mitigated through dietary interventions. To our knowledge, the effect of dietary probiotics and prebiotics on PPI‐induced gut dysbiosis is not reported in the literature.

Evidence from our lab and others indicates that dietary berries are potential prebiotic sources that promote the growth of beneficial microbes and improve gut dysbiosis in preclinical and human studies [15, 16, 17, 18, 19]. We recently demonstrated that dietary blueberries and strawberries at physiologically relevant concentrations (equivalent to 1.5 or 2 human servings) alleviate gut dysbiosis and vascular endothelial inflammation in diabetic mice and mice fed high‐fat diets [15, 16, 17]. Strawberries and blueberries are rich in phytochemicals such as anthocyanins, which consist of a carbohydrate component (glucose, galactose, arabinose) and anthocyanidin (cyanidin, delphinidin, malvidin, petunidin, peonidin, and pelargonidin). Following consumption, most anthocyanins reach the colon as human digestive enzymes cannot metabolize them. Microbial enzymes metabolize anthocyanins, and during this process, the released carbohydrate component will be utilized by the commensal microbes as an energy source. Hence, dietary berries act as a potential source of prebiotics and could provide a promising approach to preventing PPI‐induced dysbiosis and inflammation.

We investigated whether dietary blueberries or strawberries improve PPI‐induced dysbiosis and prevent gut inflammation in a preclinical model. We analyzed gut microbes in the cecum and colon contents, as the cecum is the fermentation site in animals, and the colon is the primary site for the metabolism of anthocyanins [20, 21]. We also assessed the association between gut microbes and gut inflammation.

## 2 Experimental Section

### 2.1 Experimental Animals

Male C57BL/6J mice (6 weeks old) were obtained from Jackson Laboratory (Bar Harbor, ME). The mice were housed at the University of Utah animal facility (5 mice per cage) under humane conditions with a one‐week acclimation. They were maintained in controlled artificial lighting with a 12‐hour cycle of light and dark. Environmental conditions were carefully monitored, with a constant temperature of 23°C and humidity at 45 ± 5%. The study protocol (Protocol #00002164) was approved by the Institutional Animal Care and Use Committee at the University of Utah. All procedures adhered to the guidelines outlined in the “Guide to the Care and Use of Laboratory Animals” as prescribed by the National Institutes of Health.

### 2.2 Experimental Groups

Male C57BL/6J mice (7 weeks old) were randomly assigned to four groups and given a diet containing ± omeprazole ± blueberry ± strawberry for 12 weeks. Control (C): mice received a standard diet; omeprazole (O): mice received a diet supplemented with omeprazole (40 mg/kg diet); omeprazole‐blueberry (OB): mice received a diet supplemented with omeprazole (40 mg/kg diet) and freeze‐dried blueberry powder (3.7% in diet); and omeprazole‐strawberry (OS): mice were given a diet supplemented with omeprazole (40 mg/kg diet) and freeze‐dried strawberry powder (2.35% in diet).

### 2.3 Omeprazole and Berry‐Supplemented Diets

The standard diet and diets supplemented with ± omeprazole ± blueberry ± strawberry were purchased from Research Diets Inc. (New Brunswick, NJ). In the preclinical models, especially mouse studies, 40 mg omeprazole/kg body weight is an established model to study omeprazole‐induced alterations [13, 22, 23]. In this study, we employed an already established dosage of 40 mg/kg body weight, which corresponds to 0.02% omeprazole in the diet. Although this dosage exceeds the recommended dose for humans, omeprazole has a shorter half‐life in mice compared to humans, justifying the dose in a previous study [23]. Three previous studies have utilized a similar omeprazole dosage to evaluate the effect of omeprazole on various endpoints [13, 22, 23]. The dosages of blueberry and strawberry are physiologically relevant based on our previous studies and the Food and Drug Administration's recommendation for extrapolating doses from humans to animals by normalizing body area [15, 24]. A total of 3.7% freeze‐dried blueberry powder in the diet is equivalent to 1.5 human servings (1.5 cups or 240 g) of fresh blueberries [24]. A total of 2.35% freeze‐dried strawberry powder in the diet is equivalent to 2 human servings (2 cups or 160 g) of fresh strawberries [15]. All the diets were matched for sugar (sucrose, glucose, and fructose) and fiber contents (soluble and insoluble fibers). The berry powder was integrated into the chow pellets, as was the omeprazole powder. The composition of diets is shown in Table S1.

### 2.4 Measurement of Metabolic Parameters and Collection of Samples

Experimental animals' food intake and body weight were measured weekly. Metabolic parameters were measured at the end of the 12‐week treatment period. Fasting and non‐fasting blood glucose were measured in tail vein blood samples using the Bayer Contour Next One blood glucose monitoring system (Parsippany, NJ) [15]. Body fat and lean body mass were measured by TD‐NMR using an LF50 body composition analyzer (Minispec, Bruker, Germany) [15]. At the end of 12 weeks of treatment, mice were anesthetized using 2%–5% isoflurane. The samples (blood, colon, colon contents, and cecum contents) were collected, flash‐frozen in liquid nitrogen, and stored at −80°C.

### 2.5 Microbial Community Profiling Using 16s rRNA Amplicon Sequencing

Bacterial DNA isolated from the cecum and colon contents was used to assess the effect of omeprazole and dietary berries on the gut microbiome. The bacterial DNA was isolated using the Qiagen DNeasy PowerLyzer PowerSoil kit (Qiagen, CA), as we described previously [17, 20]. Briefly, the cells were lysed physically, chemically, and enzymatically by vortexing in the PowerBead solution. The proteins, phospholipids, and other cell components were removed stepwise, retaining the DNA on a membrane via hydrophobic interactions augmented with alcohol. The DNA eluted through the membrane with nuclease‐free water and stored at −20°C. 50 ng of the bacterial DNA was used to amplify the 16S rRNA gene using forward and reverse primers (515F/806R), which are barcoded as we described previously [17]. The pooled amplicons were subjected to paired‐end sequencing (2 × 250 bp) using Illumina Miseq platform. The sequence run used approximately 30% PhiX DNA.

### 2.6 Assessment of Gut Health

The colon samples collected from the experimental mice were used to determine the effect of omeprazole and dietary berries on gut health. The markers of gut inflammation (iNOS, TNF‐α, IL‐1β, and IL‐10) and gut barrier function (MUC‐2, Cldn‐1, TJP‐1) were assessed by RT‐PCR [17, 20]. Total RNA was isolated from colon samples using the RNeasy Plus mini kit (Qiagen, CA). cDNA was synthesized using this RNA using the QuantiTect Reverse Transcription Kit (Qiagen, CA). The colon mRNA expression of markers of inflammation and gut barrier was measured by qPCR (Quantstudio 12k Flex) using specific primers (mouse) and SYBR green master mix (Thermo Fisher Scientific, MA). GAPDH was used as the control.

### 2.7 Bioinformatics and Statistical Analysis

Miseq Reporter was accomplished by demultiplexing the data, trimming the adapter, and creating the fastq files. The bioinformatics was conducted using QIIME 2 [25] and the Deblur algorithm employed to remove noise [26, 27]. A phylogenetic tree was produced with FastTree [28] Greengenes 13_8 support taxonomical ordering [29]. Rarefaction plots using the observed amplicon sequence variant were used to assess adequate sampling depth. 𝝰‐Diversity (richness, evenness, and differences between communities) was evaluated using several indices, including ASV, Shannon Diversity, Evenness, and Dominance. Weighted UniFrac differences and principal coordinate analysis were used for β‐Diversity. SPSS Version 25 (IBM) was used for the statistical calculation. R programming language (R‐4.2.2) was also used for microbial analysis.

### 2.8 Statistical Analysis

Data were analyzed using SPSS (Version 25; IBM). One‐way ANOVA analysis compared the means between groups for metabolic parameters data and the endpoints (markers of inflammation and gut barrier). The Tukey post hoc test was used if significant differences (*p* < 0.05) in main effects were found. The standard error of the mean (SEM) was used to calculate the error, and significance was determined at the 0.05 level. All data were expressed as mean value ± SEM.

## 3 Results

### 3.1 Omeprazole and Dietary Berries Did Not Alter Metabolic Parameters in Experimental Mice

The metabolic parameters were not significantly different among the experimental groups in the present study (Table 1). Supplementation of omeprazole and/or omeprazole with blueberry/strawberry supplementation did not alter body weight, food intake, body composition, or fasting and non‐fasting blood glucose in the experimental mice.

**TABLE 1:** Metabolic parameters in experimental mice.

Parameters	C	O	OB	OS
Body weight (g)	29.5 ± 0.60	29.4 ± 0.59	29.3 ± 0.52	30.4 ± 0.48
Food intake (g)	16.9 ± 0.61	16.3 ± 0.43	17.1 ± 1.09	16.6 ± 0.50
Blood glucose				
Fasting (mg/dL)	83.9 ± 2.65	79.1 ± 5.57	75.8 ± 1.76	80.8 ± 3.69
Non‐fasting (mg/dL)	148.2 ± 5.64	155.3 ± 5.76	145.1 ± 8.03	167.9 ± 4.35
Body composition				
Body fat (%)	14.9 ± 2.70	14.4 ± 1.84	14.7 ± 1.62	13.4 ± 2.85
Lean body mass (%)	70.3 ± 1.48	70.0 ± 0.73	69.7 ± 0.98	70.4 ± 1.61
Fluid (%)	12.4 ± 0.29	12.5 ± 0.32	12.4 ± 0.20	12.4 ± 0.30

Abbreviations: C: control mice; O: omeprazole‐treated mice; OB: omeprazole‐treated and blueberry‐supplemented mice; OS: omeprazole‐treated and strawberry‐supplemented mice. One‐way ANOVA analysis was used to compare the means between groups.

### 3.2 Berries Counteract Omeprazole‐Induced Alterations in Microbial Signatures in Cecum Contents

**FIGURE 1:**
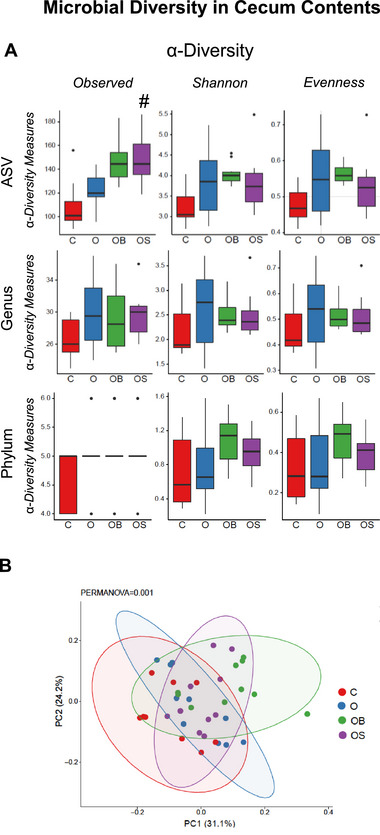
Microbial diversity in cecum contents. (A) 𝜶‐Diversity indices. (B) 𝛃‐Diversity of the microbial community. C: Mice received a standard diet; O: mice received a diet supplemented with omeprazole (40 mg/kg diet); OB: mice received a diet supplemented with omeprazole (40 mg/kg diet) and freeze‐dried blueberry powder (3.7% in diet); and OS: mice were given a diet supplemented with omeprazole (40 mg/kg diet) and freeze‐dried strawberry powder (2.35% in diet). Values are mean ± SEM (*n* = 10). ^*^ *p* < 0.05, O vs. C; ^#^ *p* < 0.05, OB vs. O or OS vs. O.

**FIGURE 2:**
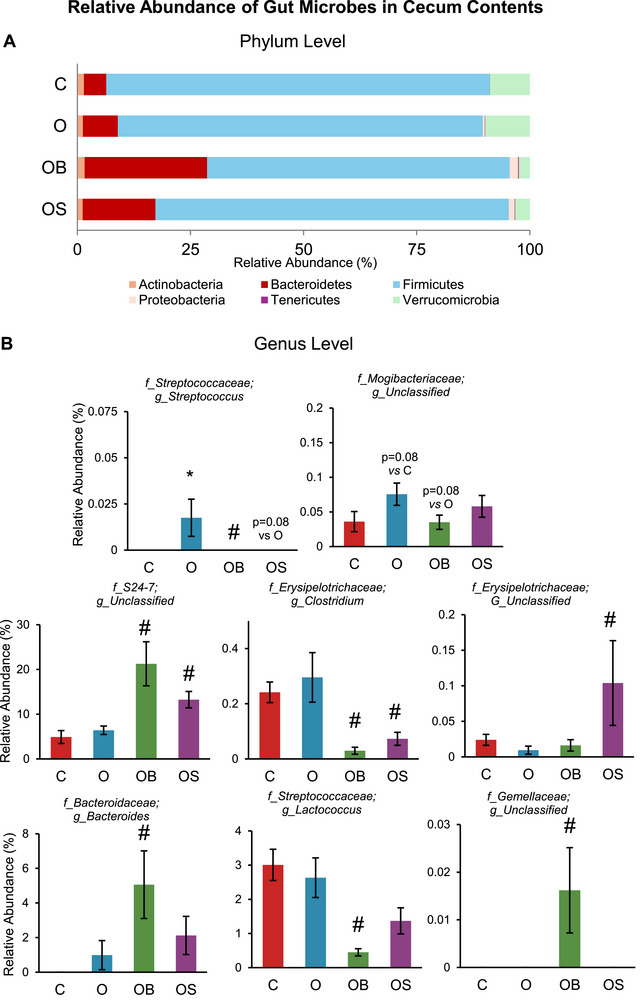
Relative abundance of gut microbes in cecum contents. (A) Phylum level microbial abundance. (B) Genus level microbial abundance. C: Mice received a standard diet; O: mice received a diet supplemented with omeprazole (40 mg/kg diet); OB: mice received a diet supplemented with omeprazole (40 mg/kg diet) and freeze‐dried blueberry powder (3.7% in diet); and OS: mice were given a diet supplemented with omeprazole (40 mg/kg diet) and freeze‐dried strawberry powder (2.35% in diet). Values are mean ± SEM (*n* = 10). ^*^ *p* < 0.05, O vs. C; ^#^ *p* < 0.05, OB vs. O or OS vs. O.

α‐Diversity indices (Observed, Shannon, and Evenness) measure the gut microbial richness and evenness. In the present study, omeprazole treatment did not alter the α‐diversity at the phylum, genus, or ASV levels (O vs. C) (Figure 1A). However, dietary strawberries increased the α‐diversity index observed in OS vs. O. β‐diversity measures global microbial composition, representing the experimental groups' similarities and differences. In the present study, omeprazole and berry supplementation impacted β‐diversity (Figure 1B). β‐diversity in omeprazole‐treated mice showed a trend (*p* = 0.088) compared to control (O vs. C). The blueberry and strawberry groups significantly differed from the omeprazole groups (OB vs. O and OS vs. O). The qualitative measure of the relative abundance of gut microbes at the phylum level is shown in Figure 2A. Bacterial sequences were distributed among six phyla, including Actinobacteria, Bacteroidetes, Firmicutes, Proteobacteria, Tenericutes, and Verrucomicrobia. The relative abundance at the phyla level was not significantly different between O vs. C. However, mice treated with blueberry showed a significant increase in Bacteroidetes and a decreasing trend in Firmicutes compared to omeprazole‐only treated mice (OB vs. O). Further, strawberries increased the abundance of Bacteroidetes compared to omeprazole‐only treated mice (OS vs. O). The relative abundance of selected genera significantly differs between the experimental groups, as shown in Figure 2B. *Streptococcus* significantly increased, and unclassified genera belonging to the family *Mogibacteriaceae* showed an increasing trend (*p* = 0.08) in omeprazole‐treated mice (O vs. C). Blueberry supplementation decreased *Streptococcus* and showed a decreasing trend (*p* = 0.08) for the unclassified genera of the family *Mogibacteriaceae* in OB vs. O. Blueberry supplementation further increased *Bacteroides*, unclassified genera belonging to the family *S24‐7* and *Gemeliaceae*, whereas decreased *Clostridium* and *Lactococcus* in OB vs. O. Strawberry supplementation significantly decreased *Clostridium*, unclassified genera belonging to the family *S24‐7* as well as increased unclassified genera belonging to the family *Erysipelotrichaceae* in OS vs. O. Strawberry supplementation showed a decreasing trend in *Streptococcus* (*p* = 0.08) in OS vs. O.

### 3.3 Berries Counteract Omeprazole‐Induced Alterations in Microbial Signatures in the Colon

**FIGURE 3:**
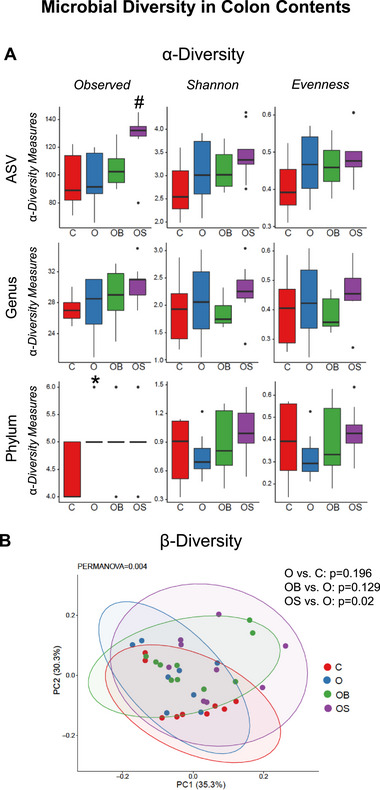
Microbial diversity in colon contents. (A) 𝜶‐Diversity indices. (B) 𝛃‐Diversity of the microbial community. C: Mice received a standard diet; O: mice received a diet supplemented with omeprazole (40 mg/kg diet); OB: mice received a diet supplemented with omeprazole (40 mg/kg diet) and freeze‐dried blueberry powder (3.7% in diet); and OS: mice were given a diet supplemented with omeprazole (40 mg/kg diet) and freeze‐dried strawberry powder (2.35% in diet). Values are mean ± SEM (*n* = 10). ^*^ *p* < 0.05, O vs. C; ^#^ *p* < 0.05, OB vs. O or OS vs. O.

**FIGURE 4:**
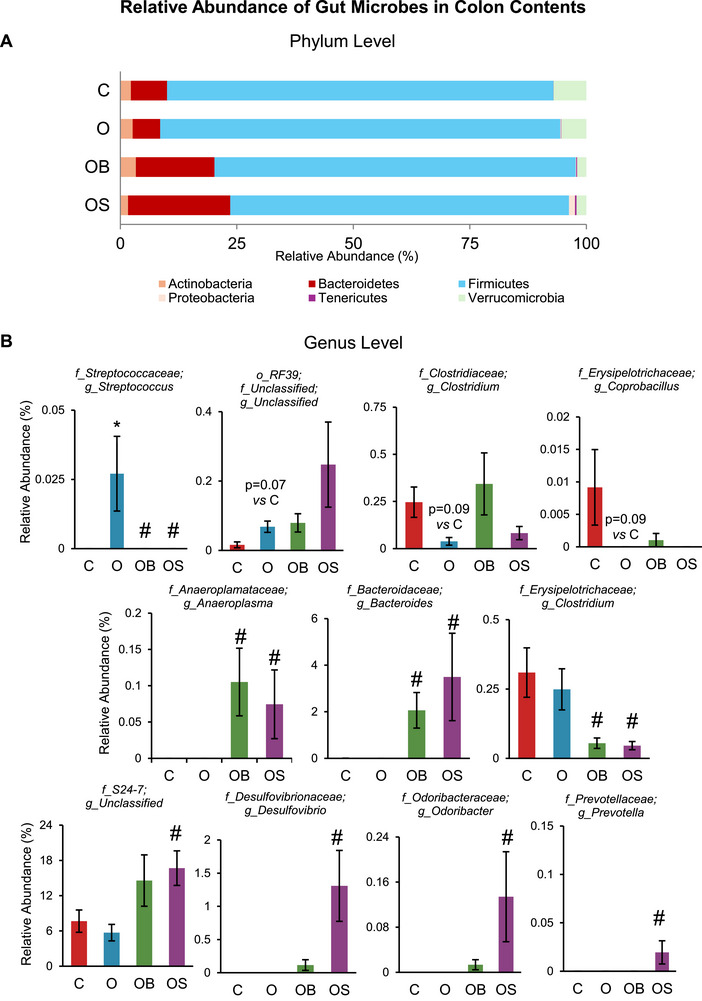
Relative abundance of gut microbes in colon contents. (A) Phylum level microbial abundance. (B) Genus level microbial abundance. C: Mice received a standard diet; O: mice received a diet supplemented with omeprazole (40 mg/kg diet); OB: mice received a diet supplemented with omeprazole (40 mg/kg diet) and freeze‐dried blueberry powder (3.7% in diet); and OS: mice were given a diet supplemented with omeprazole (40 mg/kg diet) and freeze‐dried strawberry powder (2.35% in diet). Values are mean ± SEM (*n* = 10). ^*^ *p* < 0.05, O vs. C; ^#^ *p* < 0.05, OB vs. O or OS vs. O.

The microbial signature identified in the colon is shown in Figures 3 and 4. Consistent with the cecum data, omeprazole treatment did not alter the α‐diversity at the genus or ASV levels (O vs. C) but increased the α‐diversity index (Observed) at the phylum level (Figure 3A). However, dietary strawberries increased α‐diversity in OS vs. O at the ASV level. In the present study, omeprazole and blueberry supplementation did not impact the β‐diversity of the microbial community in the colon (Figure 3B). However, strawberry supplementation significantly altered β‐diversity (OS vs. O). Bacterial sequences in the colon samples were distributed among six phyla such as Actinobacteria, Bacteroidetes, Firmicutes, Proteobacteria, Tenericutes, and Verrucomicrobia (Figure 4A). The relative abundance at the phyla level was not significantly different between O vs. C, with only Tenericutes showing an increasing trend (*p* = 0.09). Further, strawberries increased the abundance of Bacteroidetes in OS vs. O, consistent with the cecum data. In addition, it showed a decreasing trend in Verrucomicrobia (*p* = 0.06) in OS vs. O. The relative abundance of selected genera significantly differs between the experimental groups in the colon samples (Figure 4B). Consistent with cecum data, *Streptococcus* significantly increased in Omeprazole‐treated mice (O vs. C). Further, unclassified genera belonging to the order *RF39* showed an increasing trend (*p* = 0.07), whereas a decreasing trend in *Clostridium* (*p* = 0.09) and *Coprobacillus* (*p* = 0.09) in Omeprazole‐treated mice compared to control (O vs. C). Supplementation of blueberry or strawberry reversed the Omeprazole‐induced changes by decreasing the abundance of *Streptococcus* (OB vs. O and OS vs. O) Further, blueberry supplementation increased *Anaeroplasma* and *Bacteroides*, whereas decreased *Clostridium* in OB vs. O. Similarly, strawberry supplementation decreased *Clostridium*, whereas increased *Anaeroplasma* and *Bacteroides*, Unclassified genera belonging to the family *S24‐7*, *Desulfovibrio*, *Odoribacter* and *Prevotella* in OS vs. O.

### 3.4 Dietary Strawberries Suppressed Omeprazole‐Induced Increases in the mRNA Expression of iNOS

**FIGURE 5:**
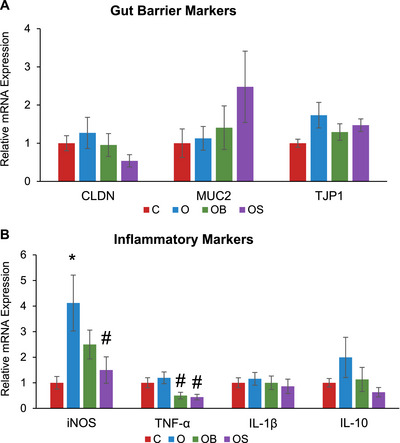
mRNA Expression of (A) gut barrier markers and (B) inflammatory markers. C: Mice received a standard diet; O: mice received a diet supplemented with omeprazole (40 mg/kg diet); OB: mice received a diet supplemented with omeprazole (40 mg/kg diet) and freeze‐dried blueberry powder (3.7% in diet); and OS: mice were given a diet supplemented with omeprazole (40 mg/kg diet) and freeze‐dried strawberry powder (2.35% in diet). Values are mean ± SEM (*n* = 10). One‐way ANOVA analysis was used to compare the means between groups and Tukey post hoc test was used if significant differences (*p* < 0.05) in main effects were found. ^*^ *p* < 0.05, O vs. C; ^#^ *p* < 0.05, OB vs. O or OS vs. O.

The mRNA expression of the markers of gut inflammation (iNOS, TNF‐α, IL‐1β, and IL‐10) and gut barrier function (MUC‐2, Cldn‐1, and TJP‐1) is shown in Figure 5. No significant differences were observed among the experimental groups for gut barrier function markers (MUC‐2, Cldn‐1, TJP‐1) and gut inflammatory markers such as IL‐1β and IL‐10. However, Omeprazole increased the mRNA expression of iNOS (O vs. C), which decreased with strawberry supplementation (OS vs. O). Both blueberry and strawberry supplementation reduced the expression of TNF‐α compared to omeprazole‐treated mice (OB vs. O and OS vs. O).

### 3.5 Selected Gut Microbes Were Associated With Inflammatory Markers

**FIGURE 6:**
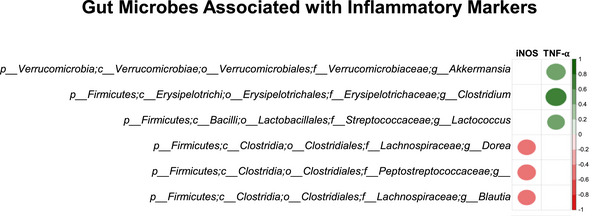
Gut microbes associated with inflammatory markers.

Spearman's correlation determined the association between gut microbes (at the genera level) and markers of inflammation (iNOS and TNF‐α that are altered between the experimental groups) (Figure 6). Genera such as *Akkermansia, Clostridium*, and *Lactococcus* were positively associated with TNF‐α. *Dorea*, an unclassified genus from the family *Peptostreptococcaceae*, and *Blautia* were negatively associated with iNOS.

## 4 Discussion

We investigated whether well‐known prebiotic dietary sources such as blueberries and strawberries ameliorate omeprazole‐induced dysbiosis and impaired gut health. First, no changes were observed in metabolic parameters in mice fed a diet supplemented with omeprazole or berries. Second, blueberry or strawberry supplementation at nutritional dosages improved alterations in gut microbial ecology induced by omeprazole, with effects varying between the cecum and colon. Third, strawberry supplementation reduced omeprazole‐induced gut inflammation. Fourth, selected genera were either positively or negatively associated with markers of gut inflammation, suggesting that dietary berries can ameliorate inflammatory signaling through modifications in the gut microbiome.

The cecum is the primary fermentation site in animals, and anthocyanins are mainly metabolized in the colon [16, 20, 21]. Hence, we analyzed the cecum and the colon contents to determine the effect of omeprazole and dietary berries on gut microbes. Human studies and preclinical studies indicate PPI‐induced alterations in the gut microbiome [30, 31]. We have recently shown that dietary supplementation of blueberries and strawberries to healthy (control) mice beneficially modulates the gut microbial composition and exhibits a prebiotic effect [15, 32]. In this study, the observed benefits could be attributed to their prebiotic effects and their specific influence on omeprazole‐induced dysbiosis. In addition, we showed blueberries and strawberries improve diabetes or high fat diet induced dysbiosis in preclinical models [17, 20, 24].

In the present study, omeprazole increased the 𝛂‐diversity index Observed at the phylum level in the colon without changing other indices. The cecum contents showed significant differences in 𝛃‐diversity in OB vs. O, OS vs. O, and a trend in O vs C. Both blueberries and strawberries ameliorated omeprazole‐induced alterations in 𝛃‐diversity in cecum samples. This indicates the impact of omeprazole, blueberries, and strawberries on the gut microbial community. However, except for OS vs. O, these changes were not observed in the colon samples. This could be due to the different microbial ecology in these two sites.

Human studies indicate that PPIs alter the abundance of gut microbes at the phylum level [33]. In our study, omeprazole did not change the abundance of gut microbes at the phylum level. However, supplementation with blueberry or strawberry increased Bacteroidetes in omeprazole‐treated mice. Furthermore, several bacteria were altered at the genus level and improved with dietary berries. Some genera showed changes in both cecum and colon contents. This includes *Bacteroides*, *Lactococcus*, *Streptococcus, Clostridium*, an unclassified genus from family *S24‐7*, and an unclassified genus of the family *Mogibacteriaceae*. Most importantly, omeprazole treatment increased the abundance of *Streptococcus*, which was reduced with blueberry or strawberry supplementation. *Streptococcus* has been identified as a biomarker of unhealthy aging, as measured by the prevalence of cancer, diabetes, cardiovascular disease, and pulmonary disease [34]. PPI treatment has previously been shown to increase the abundance of *Streptococcus* [35, 36]. Some oral‐specific species of *Streptococcaceae*, including *Streptococcus anginosus*, only survive in a pH > 5 [36]. PPIs lower the HCl content in the stomach, often raising the pH above 6 [37, 38]. Consequently*, S. anginosus*, a primarily oral species, was 42‐fold more abundant in the stool samples after PPI treatment [39]. In liver cirrhosis patients, stool abundances of oral‐specific species of *Streptococcus* were correlated to long‐term PPI use, an independent predictor of shortened survival [39]. Additionally, *Streptococcus* elevates the risk of recurrence in patients with *Clostridioides difficile infections* (CDI) [40]. In the present study, dietary blueberries and strawberries significantly reduced the abundance of *Streptococcus*. Thus, dietary berries hold the potential as a nutritive strategy to mitigate the risk of PPI‐induced complications and to enhance survival in liver cirrhosis and CDI patients. We found that blueberries and strawberries increased *Bacteroides* abundance, which is important because studies indicate that *Bacteroides* is negatively correlated with CDI recurrence and positively correlated with treatment response in infected patients [41]. Thus, berries enrich the likely commensal *Bacteroides*.

In the present study, several genera were altered only in the cecum or colon, not both. This discrepancy is likely due to their different microbial environments. In the cecum samples, omeprazole exhibited an increased trend in an unclassified genus of the family *Mogibactereaceae*, while dietary blueberries elicited a decreased trend in this genus. In a randomized controlled trial, daily consumption of orange juice for seven weeks enriched the abundance of *Mogibactereaceae* [42]. It may be that omeprazole shares a similar molecular structure or function with the components of orange juice, which promotes the growth of *Mogibactereaceae*. One possible orange component that functionally mimics omeprazole is D‐limonene. While limonene is a small ring structure and omeprazole is a large and complex molecule with multiple rings and heavy functional groups, D‐limonene is frequently used as a natural alternative to anti‐acid reflux medications [43]. Perhaps *Mogibactereaceae* metabolizes limonene as well as the smaller breakdown products of omeprazole. Blueberries may promote a competing microbe to this genus of *Mogibactereaceae*, leading to an alternative genus outcompeting it. In the colon samples, omeprazole did not affect the abundance of *Prevotella*, but strawberry supplementation increased its abundance. Studies indicate that *Prevotella* abundance diminished proportionally to the duration of daily PPI use in *C. difficile* patients [44]. While PPI use was an independent predictor of worsened mortality in CDI patients, low *Prevotella* was correlated with prolonged PPI use and predicted shortened CDI survival. The present study demonstrates that strawberries enhance *Prevotella* abundance. Therefore, a simple dietary intervention may improve CDI recovery in patients using PPI by fostering *Prevotella*.

Omeprazole treatment increased the mRNA expression of gut inflammatory marker iNOS in the colon without altering other markers such as TNF‐α, IL‐1β, and IL‐10. iNOS has served as an indicator of intestinal epithelial integrity and is upregulated in inflammatory bowel disease. iNOS activity is generally beneficial when present in tightly regulated quantities, helping to dilate blood vessels and lower blood pressure by producing nitric oxide. However, iNOS activity can shift into overdrive in chronic inflammation, as observed in inflammatory bowel disease. Inflammatory responses require blood vessels to dilate and become more permeable to release immune cells and nutrients. Thus, it makes sense that iNOS would be overly active when the gut is inflamed [45]. By synthesizing NO, iNOS provides a rich source of nitrate on which opportunistic microbes thrive. The current study found that strawberry supplementation, but not blueberry, suppressed iNOS. This discrepancy between the effects of the two different berries is likely due to their different anthocyanins. Our previous study shows strawberries are rich in pelargonidin anthocyanin (0.36 mg/kg freeze‐dried powder), while blueberries lack pelargonidin [32]. The present study suggests that dietary supplementation of strawberries could improve omeprazole‐induced gut inflammation by reducing iNOS.

Omeprazole did not increase the mRNA expression of TNF‐α in this study. However, both blueberry and strawberry supplementation decreased TNF‐α expression. TNF‐α is a key inflammatory cytokine produced by monocytes and macrophages, signaling the progression to necrosis (tissue death) and apoptosis (cell death). It is involved in several metabolic diseases such as IBD, atherosclerosis, and nonalcoholic fatty liver disease [46, 47]. In this study, both blueberry and strawberry supplementation reduced TNF‐α expression. Since berries diversify and strengthen a healthy microbiome, the commensal microbes they support may produce metabolites that inhibit TNF‐α expression. Thus, berries may be a potential nutritional strategy to help prevent chronic illnesses driven by TNF‐𝛼. In the present study, omeprazole, blueberries or strawberries did not affect the expression of gut barrier function. While mRNA levels can offer insights into gene regulation, they do not always directly correspond to protein expression or functional activity. This is a known limitation of our study, as protein‐level data would enhance the findings and give a clearer picture of the inflammatory response.

We have previously shown that blueberries or strawberries at physiologically relevant concentrations do not alter metabolic parameters in different experimental models, such as diabetic mice and high‐fat diet‐fed mice [20, 24]. In the present study, metabolic parameters such as body weight, food intake, body composition, and blood glucose were not altered with omeprazole and dietary berries. Therefore, the effect of berries on gut microbiome and inflammatory markers observed in the present study is due to gut‐targeted benefits rather than systemic metabolic benefits.

Lastly, Spearman's correlation indicated that *Akkermansia, Clostridium*, and *Lactococcus* were positively associated with TNF‐α. Furthermore, *Blautia*, *Dorea*, and an unclassified genus of the family *Peptostreptococcaceae* were negatively associated with iNOS. *Akkermansia*, which belongs to the phylum Verrucomicrobia, positively correlated with TNF‐α. *Clostridium* belongs to the *Erysipelotrichaceae* family and is positively correlated with TNF‐α. Dietary supplementation with blueberries or strawberries reduces *Clostridium levels*. Evidence suggests that lowering *Clostridium* may help prevent CDI recurrence in patients using PPIs [48]. *Lactococcus*, positively associated with TNF‐α, was significantly reduced by blueberries. These data suggest that dietary berries can ameliorate inflammatory signaling through modifications in the gut microbiome.

## 5 Conclusion

We investigated whether dietary berries could prevent PPI‐induced changes in the gut microbial ecology. In our study, blueberries and strawberries, excellent dietary prebiotic sources, alleviate PPI‐induced gut dysbiosis and inflammation. Dietary berries may serve as a potential nutritional strategy to mitigate PPI‐induced alterations in gut microbes and reduce gut inflammation. Future studies that assess the protein expression of permeability and inflammatory markers would further strengthen our findings.

## Funding

The authors have nothing to report.

## Conflicts of Interest

The authors declare no conflicts of interest.

## Supporting information




**Supporting Information file 1**: mnfr70307‐sup‐0001‐SuppMat.pdf

## Data Availability

The relevant data are provided in the article, as well as supplementary information, or are available from the corresponding author upon reasonable request. Bioinformatic tools, software versions, and parameters used in the present study are described in Section 2. Additional details regarding the code to reproduce the analyses are available upon request.

## References

[1] L. G. T. Shanika , A. Reynolds , S. Pattison , and R. Braund , “Proton Pump Inhibitor Use: Systematic Review of Global Trends and Practices,” European Journal of Clinical Pharmacology 79 (2023): 1159–1172. 37420019 10.1007/s00228-023-03534-z PMC10427555

[2] A. Delcher , S. Hily , A. S. Boureau , et al., “Multimorbidities and Overprescription of Proton Pump Inhibitors in Older Patients,” PLOS ONE 10 (2015): 0141779. 10.1371/journal.pone.0141779 PMC4633104 26535585

[3] Y. Liu , X. Zhu , R. Li , J. Zhang , and F. Zhang , “Proton Pump Inhibitor Utilisation and Potentially Inappropriate Prescribing Analysis: Insights From a Single‐Centred Retrospective Study,” BMJ Open 10 (2020): 040473. 10.1136/bmjopen-2020-040473 PMC7692833 33243802

[4] D. Beconcini , A. Fabiano , R. Di Stefano , et al., “Cherry Extract From *Prunus avium* L. to Improve the Resistance of Endothelial Cells to Oxidative Stress: Mucoadhesive Chitosan vs. Poly(lactic‐co‐glycolic acid) Nanoparticles,” International Journal of Molecular Sciences 20 (2019): 1759. 30974730 10.3390/ijms20071759 PMC6480209

[5] W. K. Lo and W. W. Chan , “Proton Pump Inhibitor Use and the Risk of Small Intestinal Bacterial Overgrowth: A Meta‐Analysis,” Clinical Gastroenterology and Hepatology 11 (2013): 483–490. 23270866 10.1016/j.cgh.2012.12.011

[6] B. R. Jagdish and W. R. Kilgore 3rd , “The Relationship Between Functional Dyspepsia, PPI Therapy, and the Gastric Microbiome,” Kansas Journal of Medicine 14 (2021): 136–140. 34084274 10.17161/kjm.vol1414831 PMC8158412

[7] J. H. Pyo , T. J. Kim , H. Lee , et al., “Proton Pump Inhibitors Use and the Risk of Fatty Liver Disease: A Nationwide Cohort Study,” Journal of Gastroenterology and Hepatology 36 (2021): 1235–1243. 32886822 10.1111/jgh.15236

[8] H. B. Assalin , K. C. G. De Almeida , D. Guadagnini , et al., “Proton Pump Inhibitor Pantoprazole Modulates Intestinal Microbiota and Induces TLR4 Signaling and Fibrosis in Mouse Liver,” International Journal of Molecular Sciences 23 (2022): 13766. 36430244 10.3390/ijms232213766 PMC9693486

[9] M. Charlot , O. Ahlehoff , M. L. Norgaard , et al., “Proton‐Pump Inhibitors Are Associated with Increased Cardiovascular Risk Independent of Clopidogrel Use,” Annals of Internal Medicine 153 (2010): 378–386. 20855802 10.7326/0003-4819-153-6-201009210-00005

[10] H. Ariel and J. P. Cooke , “Cardiovascular Risk of Proton Pump Inhibitors,” Methodist DeBakey Cardiovascular Journal 15 (2019): 214. 31687101 10.14797/mdcj-15-3-214 PMC6822659

[11] A. Horvath , F. Rainer , M. Bashir , et al., “Biomarkers for Oralization During Long‐Term Proton Pump Inhibitor Therapy Predict Survival in Cirrhosis,” Scientific Reports 9 (2019): 12000. 31427714 10.1038/s41598-019-48352-5 PMC6700098

[12] A. Trifan , C. Stanciu , I. Girleanu , et al., “Proton Pump Inhibitors Therapy and Risk of *Clostridium difficile* Infection: Systematic Review and Meta‐Analysis,” World Journal of Gastroenterology 23 (2017): 6500–6515. 29085200 10.3748/wjg.v23.i35.6500 PMC5643276

[13] Y. P. Hung , W. C. Ko , P. H. Chou , et al., “Proton‐Pump Inhibitor Exposure Aggravates *Clostridium difficile*–Associated Colitis: Evidence From a Mouse Model,” Journal of Infectious Diseases 212 (2015): 654–663. 25805751 10.1093/infdis/jiv184

[14] J. J. Heidelbaugh , “Proton Pump Inhibitors and Risk of Vitamin and Mineral Deficiency: Evidence and Clinical Implications,” Therapeutic Advances in Drug Safety 4 (2013): 125–133. 25083257 10.1177/2042098613482484 PMC4110863

[15] J. C. Miller , A. K. Satheesh Babu , C. Petersen , et al., “Gut Microbes Are Associated With the Vascular Beneficial Effects of Dietary Strawberry on Metabolic Syndrome‐Induced Vascular Inflammation,” Molecular Nutrition & Food Research 66 (2022): 2200112. 10.1002/mnfr.202200112 PMC9691581 36112603

[16] C. Petersen , U. D. Wankhade , D. Bharat , et al., “Dietary Supplementation With Strawberry Induces Marked Changes in the Composition and Functional Potential of the Gut Microbiome in Diabetic Mice,” Journal of Nutritional Biochemistry 66 (2019): 63–69. 30771735 10.1016/j.jnutbio.2019.01.004 PMC6490960

[17] A. K. Satheesh Babu , C. Petersen , H. A. Paz , et al., “Dose‐ and Time‐Dependent Effect of Dietary Blueberries on Diabetic Vasculature Is Correlated With Gut Microbial Signature,” Antioxidants (Basel) 12 (2023): 1527. 37627522 10.3390/antiox12081527 PMC10451530

[18] A. Ntemiri , T. S. Ghosh , M. E. Gheller , et al., “Whole Blueberry and Isolated Polyphenol‐Rich Fractions Modulate Specific Gut Microbes in an In Vitro Colon Model and in a Pilot Study in Human Consumers,” Nutrients 12 (2020): 2800. 32932733 10.3390/nu12092800 PMC7551244

[19] Z. Ezzat‐Zadeh , S. M. Henning , J. Yang , et al., “California Strawberry Consumption Increased the Abundance of Gut Microorganisms Related to Lean Body Weight, Health and Longevity in Healthy Subjects,” Nutrition Research 85 (2021): 60–70. 33450667 10.1016/j.nutres.2020.12.006

[20] C. Petersen , A. K. Satheesh Babu , C. M. Della Lucia , et al., “Gut Microbes Metabolize Strawberry Phytochemicals and Mediate Their Beneficial Effects on Vascular Inflammation,” Gut Microbes 17 (2025): 2446375. 39760464 10.1080/19490976.2024.2446375 PMC12931721

[21] J. Quan , G. Cai , J. Ye , et al., “A Global Comparison of the Microbiome Compositions of Three Gut Locations in Commercial Pigs With Extreme Feed Conversion Ratios,” Scientific Reports 8 (2018): 4536. 29540768 10.1038/s41598-018-22692-0 PMC5852056

[22] M. Saqui‐Salces , A. C. Tsao , M. G. Gillilland 3rd , and J. L. Merchant , “Weight Gain in Mice on a High Caloric Diet and Chronically Treated With Omeprazole Depends on Sex and Genetic Background,” American Journal of Physiology Gastrointestinal and Liver Physiology 312 (2017): G15–G23. 27810953 10.1152/ajpgi.00211.2016 PMC5283905

[23] S. Tomkovich , N. A. Lesniak , Y. Li , et al., “The Proton Pump Inhibitor Omeprazole Does Not Promote *Clostridioides difficile* Colonization in a Murine Model,” *mSphere* 4 (2019): e00693–19. 10.1128/mSphere.00693-19 PMC6887860 31748246

[24] C. Petersen , D. Bharat , U. D. Wankhade , et al., “Dietary Blueberry Ameliorates Vascular Complications in Diabetic Mice Possibly Through NOX4 and Modulates Composition and Functional Diversity of Gut Microbes,” Molecular Nutrition & Food Research 66 (2022): 2100784. 10.1002/mnfr.202100784 PMC9132135 35120277

[25] E. Bolyen , J. R. Rideout , M. R. Dillon , et al., “Reproducible, Interactive, Scalable and Extensible Microbiome Data Science Using QIIME 2,” Nature Biotechnology 37 (2019): 852–857. 10.1038/s41587-019-0209-9 PMC7015180 31341288

[26] A. Amir , D. McDonald , J. A. Navas‐Molina , et al., “Deblur Rapidly Resolves Single‐Nucleotide Community Sequence Patterns,” *mSystems* 2 (2017): e00191–16. 10.1128/mSystems.00191-16 PMC5340863 28289731

[27] N. A. Bokulich , S. Subramanian , J. J. Faith , et al., “Quality‐Filtering Vastly Improves Diversity Estimates From Illumina Amplicon Sequencing,” Nature Methods 10 (2013): 57–59. 23202435 10.1038/nmeth.2276 PMC3531572

[28] M. N. Price , P. S. Dehal , and A. P. Arkin , “FastTree 2 – Approximately Maximum‐Likelihood Trees for Large Alignments,” PLOS ONE 5 (2010): 9490. 10.1371/journal.pone.0009490 PMC2835736 20224823

[29] D. McDonald , M. N. Price , J. Goodrich , et al., “An Improved Greengenes Taxonomy With Explicit Ranks for Ecological and Evolutionary Analyses of Bacteria and Archaea,” ISME Journal 6 (2012): 610–618. 22134646 10.1038/ismej.2011.139 PMC3280142

[30] G. Bruno , P. Zaccari , G. Rocco , et al., “Proton Pump Inhibitors and Dysbiosis: Current Knowledge and Aspects to be Clarified,” World Journal of Gastroenterology 25 (2019): 2706–2719. 31235994 10.3748/wjg.v25.i22.2706 PMC6580352

[31] M. Hojo , T. Asahara , A. Nagahara , et al., “Gut Microbiota Composition Before and After Use of Proton Pump Inhibitors,” Digestive Diseases and Sciences 63 (2018): 2940–2949. 29796911 10.1007/s10620-018-5122-4 PMC6182435

[32] A. K. Satheesh Babu , C. Petersen , L. Iglesias‐Carres , et al., “Blueberry Intervention Mitigates Detrimental Microbial Metabolite Trimethylamine N‐Oxide by Modulating Gut Microbes,” Biofactors 50 (2024): 392–404. 37921575 10.1002/biof.2014 PMC11014767

[33] J. H. Lim , J. Shin , and J. S. Park , “Effect of a Proton Pump Inhibitor on the Duodenum Microbiome of Gastric Ulcer Patients,” Life (Basel) 12 (2022): 1505. 36294939 10.3390/life12101505 PMC9605190

[34] H. Singh , M. G. Torralba , K. J. Moncera , et al., “Gastro‐Intestinal and Oral Microbiome Signatures Associated With Healthy Aging,” Geroscience 41 (2019): 907–921. 31620923 10.1007/s11357-019-00098-8 PMC6925087

[35] F. Imhann , A. Vich Vila , M. J. Bonder , et al., “The Influence of Proton Pump Inhibitors and Other Commonly Used Medication on the Gut Microbiota,” Gut Microbes 8 (2017): 351–358. 28118083 10.1080/19490976.2017.1284732 PMC5570416

[36] F. Paroni Sterbini , A. Palladini , L. Masucci , et al., “Effects of Proton Pump Inhibitors on the Gastric Mucosa‐Associated Microbiota in Dyspeptic Patients,” Applied and Environmental Microbiology 82 (2016): 6633–6644. 27590821 10.1128/AEM.01437-16 PMC5086557

[37] D. E. Freedberg , B. Lebwohl , and J. A. Abrams , “The Impact of Proton Pump Inhibitors on the Human Gastrointestinal Microbiome,” Clinics in Laboratory Medicine 34 (2014): 771–785. 25439276 10.1016/j.cll.2014.08.008 PMC4254461

[38] R. Rosen , L. Hu , J. Amirault , et al., “16S community Profiling Identifies Proton Pump Inhibitor Related Differences in Gastric, Lung, and Oropharyngeal Microflora,” Journal of Pediatrics 166 (2015): 917–923. 25661411 10.1016/j.jpeds.2014.12.067 PMC4380592

[39] X. Xiao , X. Zhang , J. Wang , et al., “Proton Pump Inhibitors Alter Gut Microbiota by Promoting Oral Microbiota Translocation: A Prospective Interventional Study,” Gut 73 (2024): 1098–1109. 38267200 10.1136/gutjnl-2023-330883

[40] J. R. Allegretti , J. Marcus , M. Storm , et al., “Clinical Predictors of Recurrence After Primary *Clostridioides difficile* Infection: A Prospective Cohort Study,” Digestive Diseases and Sciences 65 (2020): 1761–1766. 31667694 10.1007/s10620-019-05900-3 PMC8630805

[41] S. Khanna , E. Montassier , B. Schmidt , et al., “Gut Microbiome Predictors of Treatment Response and Recurrence in Primary *Clostridium difficile* Infection,” Alimentary Pharmacology & Therapeutics 44 (2016): 715–727. 27481036 10.1111/apt.13750 PMC5012905

[42] E. Brasili , N. M. A. Hassimotto , F. Del Chierico , et al., “Daily Consumption of Orange Juice From *Citrus sinensis* L. Osbeck Cv. Cara Cara and Cv. Bahia Differently Affects Gut Microbiota Profiling as Unveiled by an Integrated Meta‐Omics Approach,” Journal of Agricultural and Food Chemistry 67 (2019): 1381–1391. 30644740 10.1021/acs.jafc.8b05408

[43] R. M. Schulz , N. K. Ahuja , and J. L. Slavin , “Effectiveness of Nutritional Ingredients on Upper Gastrointestinal Conditions and Symptoms: A Narrative Review,” Nutrients 14 (2022): 672. 35277031 10.3390/nu14030672 PMC8839470

[44] C. Y. Lin , H. T. Cheng , C. J. Kuo , et al., “Proton Pump Inhibitor‐Induced Gut Dysbiosis Increases Mortality Rates for Patients With *Clostridioides difficile* Infection,” Microbiology Spectrum 10 (2022): 0048622. 10.1128/spectrum.00486-22 PMC9430933 35863023

[45] G. Kolios , V. Valatas , and S. G. Ward , “Nitric Oxide in Inflammatory Bowel Disease: A Universal Messenger in an Unsolved Puzzle,” Immunology 113 (2004): 427–437. 15554920 10.1111/j.1365-2567.2004.01984.x PMC1782592

[46] H. Chedotal , D. Narayanan , K. Povlsen , et al., “Small‐Molecule Modulators of Tumor Necrosis Factor Signaling,” Drug Discovery Today 28 (2023): 103575. 37003513 10.1016/j.drudis.2023.103575

[47] R. F. Souza , M. A. F. Caetano , H. I. R. Magalhaes , and P. Castelucci , “Study of Tumor Necrosis Factor Receptor in the Inflammatory Bowel Disease,” World Journal of Gastroenterology 29 (2023): 2733–2746. 37274062 10.3748/wjg.v29.i18.2733 PMC10237104

[48] K. M. D'Silva , R. Mehta , M. Mitchell , et al., “Proton Pump Inhibitor Use and Risk for Recurrent *Clostridioides difficile* Infection: A Systematic Review and Meta‐Analysis,” Clinical Microbiology and Infection 27 (2021): 697–703. 10.1016/j.cmi.2021.01.008 33465501

