# Supplementary material for: Dietary Prebiotics Modulate Omeprazole‐Induced Alterations in the Gut Microbial Signature
Source: Mol Nutr Food Res. 2025 Oct 30;69(24):e70307. doi: 10.1002/mnfr.70307 (PMC12700043; doi:10.1002/mnfr.70307)
Supplement: Supplementary file 1 — Supporting Information file 1: mnfr70307‐sup‐0001‐SuppMat.pdf [file MNFR-69-e70307-s001.pdf]

Supplementary Table

Rodent diets supplemented with ± 0.02% omeprazole ± 3.7% freeze-dried blueberry powder ± 2.35% freeze-dried strawberry powder

	Control Diet	Diet supplemented with 0.02% omeprazole	Diet supplemented with 0.02% omeprazole and 3.7% freeze-dried blueberry powder	Diet supplemented with 0.02% omeprazole and 2.35% freeze-dried blueberry powder
	**C**	**O**	**OB**	**OS**
**Ingredients (g)**				
Casein	200	200	199.2	198.1
L-Cystine	3	3	3	3
Corn Starch	496.75	496.75	488.5	491.4
Maltodextrin 10	107.3	107.3	107.3	107.3
Sucrose	68.8	68.8	68.8	68.4
Fructose	13.5	13.5	0	7.4
Dextrose	13.1	13.1	0	7.7
Cellulose	48.4	48.4	43.9	44.9
Inulin	2	2	0.4	0
Soybean Oil	0	0	0	0
Corn Oil	25	25	25	25
Lard	20	20	18.5	17.2
Mineral Mix S10026	10	10	10	10
DiCalcium Phosphate	13	13	13	13
Calcium Carbonate	5.5	5.5	5.5	5.5
Potassium Citrate, 1 H2O	16.5	16.5	16.5	16.5
Vitamin Mix V10001	10	10	10	10
Choline Bitartrate	2	2	2	2
Freeze Dried Blueberry Powder	0.0	0.0	39.0	0.0
Freeze Dried Strawberry Powder	0.0	0.0	0.0	24.7
Omeprazole	0.0	0.21	0.21	0.21
FD&C Yellow Dye #5	0	0	0	0
FD&C Red Dye #40	0	0	0	0
FD&C Blue Dye #1	0	0	0	0
**Total**	**1054.85**	**1055.06**	**1050.81**	**1052.31**
Freeze Dried Blueberry Powder (%)	0	0	3.7	0
Freeze Dried Strawberry Powder (%)	0	0	0	2.35
Omeprazole (%)	0	0.02	0.02	0.02
**Ingredients (g)**				
Protein	203.0	203.0	203.0	203.0
Carbohydrate	710.2	710.2	710.2	710.3
Fat	45.0	45.0	45.0	45.0
Soluble Fiber	2.0	2.0	2.0	2.0
Insoluble Fiber	48.4	48.4	48.4	48.4
Sucrose	68.8	68.8	68.8	68.8
Fructose	13.5	13.5	13.5	13.5
Dextrose	13.1	13.1	13.1	13.1
**Ingredients (g%)**				
Protein	19.3	19.2	19.3	19.3
Carbohydrate	67.6	67.3	67.6	67.6
Fat	4.3	4.3	4.3	4.3
Fiber	4.6	4.6	4.6	4.6
**Energy from nutrients (kcal)**				
Protein	812	812	812	812
Carbohydrate	2841	2840.8	2841	2841
Fat	405	405	405	405
Total	**4058**	**4058**	**4058**	**4058**
**Energy from nutrients (kcal%)**				
Protein	20.0	20.0	20.0	20.0
Carbohydrate	70.0	70.0	70.0	70.0
Fat	10.0	10.0	10.0	10.0
Total	100.0	100.0	100.0	100.0
